# Multi-institution longitudinal apparent diffusion coefficient measurements in a diffusion weighted imaging phantom at room temperature

**DOI:** 10.1016/j.phro.2025.100814

**Published:** 2025-07-22

**Authors:** Chris Moore, Charlotte Bull, Angela Darekar, Daniel Wilson, Alex Goodall, Prakash Manoharan, Peter Hoskin, Marcel van Herk, David L. Buckley, Damien J. McHugh, Anubhav Datta, Michael J. Dubec

**Affiliations:** a Christie Medical Physics and Engineering, The Christie NHS Foundation Trust, Manchester, UK; b Division of Cancer Sciences, The University of Manchester, Manchester, UK; c Medical Physics Department, University Hospitals of Leicester NHS Trust, Leicester, UK; d Department of Medical Physics, University Hospital Southampton NHS Foundation Trust, Southampton, UK; e Department of Medical Physics and Engineering, Leeds Teaching Hospitals NHS Trust, Leeds, UK; f MR Physics, Sheffield Teaching Hospital NHS Foundation Trust, Sheffield, UK; g Clinical Radiology, The Christie NHS Foundation Trust, Manchester UK; h Clinical Oncology, Mount Vernon Cancer Centre, Northwood, UK; i Biomedical Imaging, University of Leeds, Leeds, UK

**Keywords:** Quantitative apparent diffusion coefficient, QA, Imaging biomarkers, Technical validation, Multi-centre longitudinal QA

## Abstract

**Background and purpose:**

This work contributes to technical validation of apparent diffusion coefficient (ADC) as a biomarker of cancer. The aim was to evaluate ADC accuracy, random error, short-term and long-term repeatability and reproducibility, across multiple institutions using a room temperature phantom.

**Materials and methods:**

ADC measurements were made in a travelling room temperature diffusion weighted imaging (DWI) phantom on six scanners at four UK institutions over 18 months at six-month intervals. ADC bias measurements were calculated as the difference between measured and temperature corrected ground-truth ADC values and used to calculate mean ADC bias, isocentre ADC error estimate, short- and long-term intra-scanner repeatability as per the Quantitative Imaging Biomarkers Alliance (QIBA) DWI profiles, and inter-scanner reproducibility by calculating the 95 % limits of agreement for all ADC bias measurements.

**Results:**

The use of a room-temperature phantom with a magnetic resonance (MR) readable thermometer enabled ADC measurements without ice-water setup, considerably simplifying logistics with respect to multi-institution ADC quality assurance. Mean ADC bias across all scanners and sessions was <0.01 × 10^−3^ mm^2^ s^−1^ (0.81 %); mean isocentre ADC error estimate was 1.43 %; average scanner short-term repeatability was <0.01 × 10^−3^ mm^2^ s^−1^ (1 %). Reproducibility was 0.07 × 10^−3^ mm^2^ s^−1^ (9 %).

**Conclusion:**

Results indicated good ADC accuracy, repeatability and reproducibility; demonstrating the feasibility of transferring diagnostic DWI sequences between scanners from the same manufacturer, for use in multi-institution longitudinal studies, and assessing ADC with minimal quality control and harmonisation steps required.

## Highlights

• ADC was measured on six MR scanners at four institutions over 18 months.
• A room temperature DWI phantom was used.
• 95 % limits of agreement for ADC bias for all scans was 0.07 × 10^−3^ mm^2^ s^−1^ (9 %).
• We found good ADC accuracy, short and long-term repeatability and reproducibility.
• All scanners met the QIBA requirements for ADC bias, error and repeatability.

## 1 Introduction and motivation

There is a need to develop imaging biomarkers for cancer to monitor treatment response and enable stratification for treatment personalisation [1]. Many potential imaging biomarker candidates have been identified in the research setting, but few have been translated into the clinic due to incomplete technical or clinical validation [2].

Diffusion weighted imaging (DWI) is a magnetic resonance imaging (MRI) technique used to assess the diffusion of water molecules in tissue, with highly cellular tissues such as tumour showing restricted water diffusion [3]. DWI is commonly used in clinical practice for multiple tumour sites, including cervical cancers [[4], [5], [6]]. Apparent Diffusion Coefficient (ADC) maps, derived from DWI images, are commonly used qualitatively, but the quantitative ADC parameters obtained are seldom used to define thresholds, e.g. for predicting treatment response, or treatment adaption. To permit translation of quantitative ADC into routine clinical practice, performance of the measurements must be validated [2]. Important requirements for imaging biomarkers, including ADC, are that measurements are repeatable and reproducible [2]. Standardisation of sequences is an important way to achieve this by reducing measurement variability between sites and across time [2]. The Quantitative Imaging Biomarkers Alliance (QIBA) developed a set of DWI sequence profiles to aid standardisation of quantitative ADC measurement [7]. These contain standards the sequences should meet to be considered acceptable for measuring quantitative ADC, which we can use to benchmark other sequences.

Previous work by van Houdt et al. assessed quantitative ADC measurement repeatability and reproducibility at multiple institutions, with scanners from different vendors, using an ice-water diffusion phantom for a benchmark sequence meeting the QIBA profile and local institutional sequences; finding poorer repeatability and reproducibility with the institutional sequences [8]. The study assessed short-term repeatability from measurements obtained at a single session for each scanner but did not consider long-term reproducibility. For patients undergoing treatment where quantitative imaging biomarkers will be monitored; patients are likely to have imaging before, throughout and after treatment (including follow-up) over months to years; therefore, it is important that measurements are repeatable and reproducible over such time periods.

Furthermore, due to using the ice-water phantom, it was not possible to measure ADC values higher than 1.1 × 10^−3^ mm^2^ s^−1^ (i.e. the ADC of pure water at 0 °C) [8]. ADC measurements as high as 1.5 × 10^−3^ mm^2^ s^−1^ and 1.7 × 10^−3^ mm^2^ s^−1^ have been measured in prostate and cervix tissue, respectively [[9], [10], [11], [12]]. Room temperature DWI phantoms permit ADC measurements across a wider range of ADC values and permit assessment of repeatability and reproducibility over this range [13]. Additionally, the logistics of using such a phantom are much simplified, as the requirement to prepare the ice-water bath before scanning is removed.

The motivation for this work was to assess longitudinal scanner ADC performance at multiple institutions in anticipation of a study measuring quantitative ADC changes in the cervix in a multi-centre clinical trial [14], with this work constituting technical validation of the proposed DWI sequence.

The aim of this study was to assess accuracy, random measurement error, longitudinal repeatability and reproducibility of quantitative ADC measurements, across multiple institutions, using a room temperature phantom.

## 2 Materials and methods

### 2.1 MR scanners and protocol

Six Siemens 1.5 T MRI scanners at four institutions across the UK were included in the study (Table 1). The clinical oncology cervix DWI sequence (Table 2) used for diagnosis and staging at Institution A was transferred electronically to the other scanners using the vendor proprietary format to ensure consistency. Full sequence parameters and files are available upon request.

**Table 1:** Scanner information.

Study scanner ID	Study institution ID	Scanner model	Receive coils	software version
1	A	Siemens Aera	Body-18 + posterior	E11 (visit 1), XA30 (visits 2–4)
2	A	Siemens Aera	Body-18 + posterior	E11 (visit 1), XA30 (visits 2–4)
3	B	Siemens Sola	Body-30 + posterior	XA51 (visits 1–4)
4	B	Siemens Aera	Body-30 + posterior	E11 (visits 1–4)
5	C	Siemens Avanto Fit	Body-18 + posterior	E11 (visits 1–4)
6	D	Siemens Aera	Body-18 + posterior	E11 (visits 1–4)

**Table 2:** Clinical cervix DWI sequence parameters.

Sequence parameter	Parameter value
Sequence type	Single-shot echo-planar image (SS-EPI)
Orientation	Axial
In-plane field of view [mm]	240 × 206
Acquired matrix size	128 × 128
In-plane resolution (acquired/reconstructed) [mm]	1.9 × 1.6/1.0 × 1.0
Phase encode direction	AP
Slice thickness/spacing [mm]	4/0.4
Number of slices	24
TR/TE [ms]	4900–5000/71
B-values (averages) [s/mm^2^]	50 (2), 400 (2), 800 (5), 1000 (7)
Diffusion encoding scheme	4-scan trace
Receiver bandwidth [Hz/pixel]	1240
Fat-saturation	SPAIR
Parallel imaging (GRAPPA) factor	2
EPI factor	108
Partial Fourier factor	6/8
Phase oversampling	50 %
AI reconstruction	None
Acquisition time	5:40

### 2.2 Phantom

**Fig. 1:**
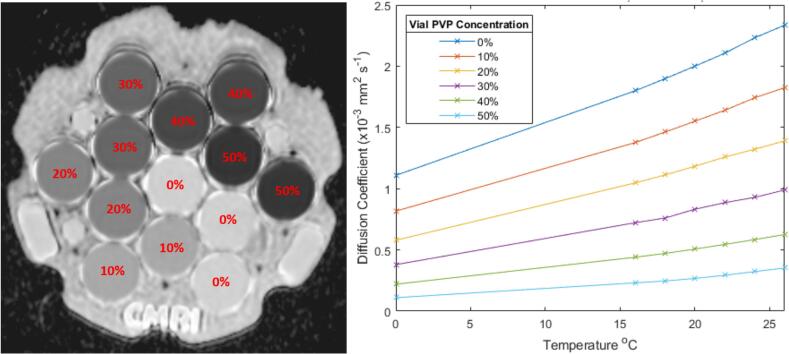
ADC map showing vial PVP concentration distribution (left) and vial diffusion coefficient dependence on temperature and vial PVP concentration (right) [reproduced from phantom calibration data].

A diffusion phantom (Model 128, CalibreMRI) was used to perform ADC measurements. The phantom is similar to the ice-water phantom used by van Houdt et al. [8] with the addition of an MR-readable thermometer. The phantom consists of 13 vials filled with different concentrations of water-polyvinylpyrrolidone (PVP) solution, from 0 to 50 % PVP. Three vials contain 0 % PVP, and two vials each contain 10, 20, 30, 40 and 50 % PVP concentration [13]. As ADC depends on PVP concentration [15], this results in six different ground-truth ADCs (Fig. 1). The bulk space is filled with water. The phantom contains an MR-readable liquid crystal (LC) thermometer [16]. This consists of 10 LC elements which transition at a particular temperature, above which they give high signal on T_1_-weighted (T_1_w) images. This allows measurement of temperature from 15 to 24 °C in approximately 1 °C increments.

Calibration certificates for the LC thermometer element temperature transition values and vial diffusion coefficients at a range of temperatures, performed by the National Institute of Standards and Technology (NIST), were provided by the phantom manufacturer.

### 2.3 Phantom scanning

The DWI phantom was imaged on the six scanners at four separate sessions, approximately six months apart (mean = 5.9 months; min = 5.5 months, max = 7.5 months) from April 2023 to December 2024.

The same phantom was used for all scans, travelling between institutions. Due to logistical limitations, it was not always possible to allow the phantom temperature to equilibrate to the magnet room.

At each session, the phantom was positioned with the long axes of the vials aligned with the scanner bore, with the centre vial at isocentre. The receive coils used were those that the institution would use clinically to scan patients with cervical cancer (Table 1).

First, a 3D T_1_-weighted (T_1_w) spoiled gradient-echo sequence was acquired to obtain the initial phantom temperature. Next, four acquisitions of the DWI sequence were obtained, with the last three acquisitions inheriting the shim settings from the first acquisition. Finally, the 3D T_1_w gradient-echo sequence was repeated to obtain the final phantom temperature [13].

### 2.4 Image analysis

ADC maps were generated from the diffusion-weighted images in MATLAB R2024 (Mathworks, Nantick MA) by voxel-wise linear least-squares fitting of the natural log of signal intensity from corresponding voxels in the different diffusion-weighted images as a function of acquired b-values. Circular regions of interest (ROI), radius 5 mm, were placed over the PVP vials in the centre slice and extended four slices superior and inferior to provide cylindrical volume ROIs for each vial. The measured ADC for each vial was calculated as the median value of the respective vial ROI.

The phantom temperature was obtained at the start and end of scanning using the T_1_w sequence, and finding the highest bright LC vial and lowest dark LC vial and calculating the mean of their corresponding transition temperatures. This temperature was then used to calculate the temperature-corrected ground-truth ADC in the vials from a plot generated by linearly interpolating the calibration certificate data points, over the temperature range of 15 °C to 26 °C (Fig. 1). If the temperature changed during scanning, the temperature for each acquisition was linearly interpolated across time, between the initial and final temperatures, assuming the temperature changed at a constant rate.

### 2.5 Statistical analysis

Statistical analysis was performed in MATLAB R2024 (Mathworks, Nantick MA).

ADC bias (ADC_bias_) was calculated as the difference between measured ADC (ADC_measured_) and temperature-corrected ground-truth ADC (ADC_TCGT_) for each vial at each acquisition:

(1) $$\mathrm{AD}C_{\mathrm{bias}}=ADC_{\mathrm{measured}}-ADC_{\mathrm{TCGT}}$$

To evaluate uncertainty of ADC_bias_ measurements due to temperature uncertainties, the range of ADC values resulting from the ∼1 °C temperature resolution at a given temperature for each of the PVP vial concentrations were calculated from the calibration certificate (Fig. 1) and plotted against temperature, assuming a linear relationship over the temperature range.

Due to temperature dependence of ground-truth ADC, accuracy, repeatability and reproducibility were assessed using ADC_bias_, rather than measured ADC values.

Kendall’s Tau test was carried out on all data from all scanners at all sessions to determine if the magnitude of ADC_bias_ was proportional to the ground-truth measurement, to identify if absolute or percentage biases should be used [17].

ADC_bias_ measurements were plotted against ADC_TCGT_ from the four sessions for each scanner and all scanners combined. The mean difference and 95 % limits of agreement were calculated for each scanner, and all scanners combined [18]. This was repeated for ADC_bias_ as a percentage of ADC_TCGT_.

The ADC error estimate for the central vial was calculated according to the QIBA profiles:

(2) $$\mathrm{Isocentre}\;ADC\;Error\;Estimate=100\times \frac{\mu_{\mathrm{Central}\;ROI}}{\sigma_{\mathrm{Central}\;ROI}}\%$$

where *µ_central ROI_* and *σ_central ROI_* are the voxel mean and standard deviation respectively for the central vial ROI [7,8]. This value was calculated for each acquisition across all sessions and used to calculate a mean isocentre ADC random error estimate, with associated standard error [19], for each scanner and all scanners combined.

To calculate scanner short-term repeatability, the 95 % session repeatability (RC_session_) was first calculated [20]:

(3) $$RC_{\mathrm{session}}=2.77\times \;\frac{1}{n_{\mathrm{vials}}}\sum_{\mathrm{vials}}\sigma_{\mathrm{vial},\;\;session}$$

where σ_vial,session_ is the standard deviation of the four ADC_bias_ measurements within the session, for a given vial and n_vials_ is the number of vials in the phantom (thirteen in this phantom). The short-term repeatability coefficient (RC_short_) for each scanner was then calculated as the average RC_session_ across the sessions:

(4) $$RC_{\mathrm{short}}=\;\frac{1}{n_{\mathrm{sessions}}}\sum_{\mathrm{sessions}}RC_{\mathrm{session}}$$

where n_sessions_ is the number of sessions (four in this study).

To provide a measure of the long-term reproducibility, 95 % limits of agreement were calculated from the ADC_bias_ from all scanners and sessions

(5) $$RC_{\mathrm{long}}=\;\frac{1}{n_{\mathrm{all}}}\sum_{\mathrm{all}}ADC_{\mathrm{bias}}\pm 1.96\times \sigma_{\mathrm{all}}$$

where *n_all_* and *σ_all_* the total number and standard deviation of ADC_bias_ measurements across all vials, sessions and scanners – i.e. 95 % of all ADC_bias_ measurements made in the study were within these limits.

For this study, ADC_bias_, RC_short_ and RC_long_ were calculated as averages across all vials to better represent the clinical range of ADC and to account for inter-session temperature differences.

## 3 Results

The DWI sequence was successfully transferred to each of the six scanners, across the four institutions. Due to hardware and software differences, the TR varied slightly (Table 2) but was deemed of insignificant impact due to the long TR. All other parameters were consistent across scanners.

**Fig. 2:**
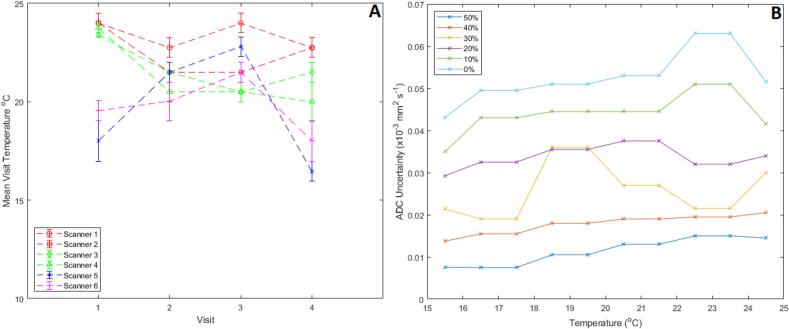
(A) Plot illustrating the temperature at each session. The points indicate the mean session temperature, and the error bars represent the possible range of temperatures in the session due to the combination of the temperature resolution and the possible change in temperature throughout the session (i.e the lowest temperature measured in the session – 0.5 to the highest temperature measured in the session +0.5). (B) Ground truth diffusion coefficient uncertainty for each vial PVP concentration as a function of temperature as a result of the temperature resolution, derived from the phantom calibration certificate.

The temperature range for all sessions was 17 to 24 °C (Fig. 2A). Scanner 5 had the largest inter-session temperature variability, while scanners 4, 5, and 6 experienced intra-session temperature variations of 1 °C during some of their sessions. Uncertainties related to temperature resolution are greater for lower PVP concentration/higher ADC vials (Fig. 2B).

There was no significant dependence of ADC_bias_ on ADC_TCGT_ (p = 0.06, Kendall Tau). As such, subsequent analysis focussed on absolute differences.

**Fig. 3:**
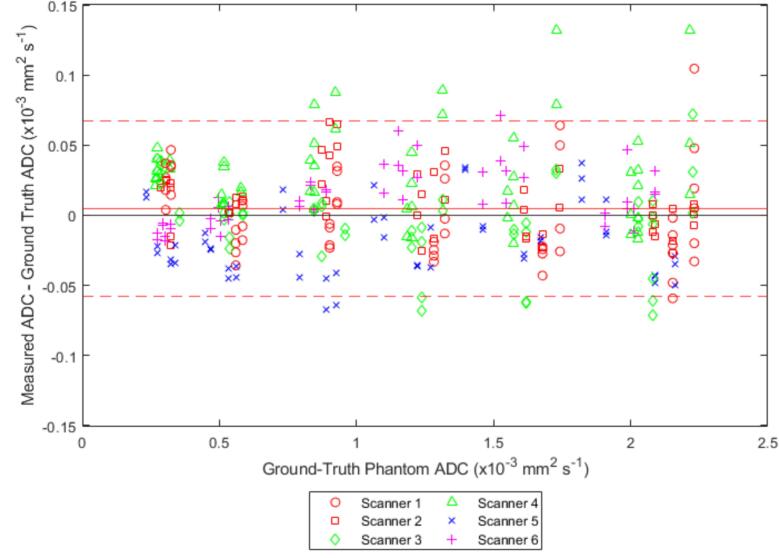
ADC bias plot for all scanner sessions. Solid red line indicates the mean bias and dashed red lines indicate 95% limits of agreement.

Across all scanners, the mean [95 % CI] ADC_bias_ was 0.00 [–0.06 to 0.07] × 10^−3^ mm^2^ s^−1^ (Table 3, Fig. 3). Fig. S7 shows the same results as percentages. The mean ADC_bias_ for each scanner is reported in Table 3 and Bland-Altman plots with 95 % limits of agreement are shown for each system in the supplementary material (Figs. S1 to S6) [7]. Isocentre ADC error estimate for each scanner and the mean value across all scanners are given in Table 3. RC_short_ was 0.01 × 10^−3^ mm^2^ s^−1^, and the greatest measured RC_short_ was scanner 6 with 0.014 × 10^−3^ mm^2^ s^−1^ [7]. Fig. S8 shows the contributions of each vial to the RC_short_ of each scanner. The 95 % limits of agreement on the ADC_bias_ plots for all scanners and sessions give a measure of reproducibility. These were measured to be −0.06 to 0.07 × 10^−3^ mm^2^ s^−1^ or −7.8 to 8.6 %.

**Table 3:** Summary numerical results for the ADC bias, average ADC error and short-term repeatability coefficient calculations for each scanner and all scanners combined.

Scanner	Institution	Bias ADC [95 % LOA] (×10^−3^ mm^2^ s^−1^)	Bias ADC [95 % LOA] (%)	Isocentre ADC Error Estimate [standard Error] (%)	Short Term RC [95 % CI] (×10^−3^ mm^2^ s^−1^)	Short-Term%RC [95 % CI] (%)
1	A	<0.01 [−0.06 to 0.06]	0.84 [−7.5 to 9.2]	1.09 [1.06 to 1.13]	0.0095 [0.0081 – 0.011]	0.98 [0.63 to 1.3]
2		0.01 [−0.03 to 0.05]	1.5 [ −4.8 to 6.3]	1.00 [0.98 to 1.02]	0.0090 [0.0070 to 0.011]	0.80 [0.68 to 0.91]
3	B	−0.01 [−0.06 to 0.05]	0.64 [−8.1 to 9.5]	1.00 [0.98 to 1.02]	0.0064 [0.0052 to 0.0076]	0.73 [0.35 to 1.1]
4		0.03 [−0.04 to 0.1]	4.0 [−4.8 to 12.8]	1.06 [1.00 to 1.11]	0.0099 [0.0077 to 0.0121]	1.4 [0.59 to 2.1]
5	C	0.01 [−0.03 to 0.05]	0.46 [−4.5 to 5.4]	1.10 [1.08 to 1.12]	0.0046 [0.0038 – 0.0053]	0.73 [0.51 to 0.96]
6	D	−0.02 [−0.07 to 0.03]	−2.7 [−10 to 5.1]	0.91 [0.88 to 0.95]	0.014 [0.011 – 0.017]	1.3 [1.0 to 1.6]
**Overall**		**<0.01** [−**0.06 to 0.07]**	**0.81** [−**7.8 to 8.6]**	**1.02** **[0.96 to 1.10]**	**<0.01** **[0.0063 – 0.011]**	**0.98** **[0.76 to 1.2]**

## 4 Discussion

All scanners met the requirements of the QIBA profile for isocentre ADC error estimate (Isocentre ADC error estimate < 2 %) and mean ADC_bias_ bias across all vials (|mean ADC Bias| <0.04 × 10^−3^ mm^2^ s^−1^) [7].

The requirement of the QIBA profile is RC_short_ < 0.015 × 10^−3^ mm^2^ s^−1^ [7]. All scanners, individually and combined, met this requirement, although the upper 95 % confidence limit for scanner 6 (0.017 × 10^−3^ mm^2^ s^−1^) fell slightly outside this.

Reproducibility measured in this study was lower than that by van Houdt et al. [8] of 18 %.

Grech-Sollars et al. measured multi-centre reproducibility of ADC of water at 0 °C, finding a coefficient of variation of 1.5 % which corresponds to a 95 % RC of 4.2 % [21] Similarly, Malyarenko et al. measured day-to-day repeatability of ADC of water at 0 °C on a range of scanners to be within 4.5 % [22]. These results agree with the reproducibility of the measurements of ADC for the central vials (the highest ADC points in Fig. 3/Fig. S7).

We were successful in setting up a standardised DWI sequence across multiple scanners and institutions, with minimal changes in sequence parameters. Furthermore, the ADC_bias_ and RC_short_ were comparable to the work by van Houdt et al. but ADC error measurements in our study were better (up to 1.1 % vs up to 3 %), likely due to standardisation of sequences and the use of a single scanner manufacturer, at the same field strength (1.5 T only) [8]. In our study, we used a room temperature DWI phantom, simplifying logistics by removing the ice-water setup at each session, which is impractical in a travelling phantom study. However, inter-session temperature variation was a limitation, as was the 1 °C temperature resolution. Since ground truth ADCs vary with temperature, different ADC measurements are effectively taken at each session. While this can be accounted for, it complicates assessments of long-term repeatability and reproducibility. This could have been improved if the phantom could have been left for long enough for the temperature to equilibrate with the magnet room; even so, variations in magnet room temperature between sessions and sites would hamper absolute ADC comparisons. In contrast, van Houdt et al. [8] consistently measured the phantom at 0 °C, thereby ensuring identical ground truth ADC values and so enabling more direct comparisons between scanners.

Measurements were performed using a phantom, which was required to provide reliable known ground-truth measurements throughout the course of the study, However, it should be noted that phantom images typically have higher SNR than is achievable in-vivo, which limits applicability of our results to patient studies as low SNR can introduce bias to ADC measurements [23]. Additionally, the phantom was a free-diffusion phantom which cannot simulate perfusion effects, which are significant at low b-values, and again introduce bias to ADC measurements [3]. However, in-vivo factors which affect ADC may be of interest and detectable by the sequence, and in-vivo perfusion effects could be assessed by fitting ADC to different b-values or mitigated by fitting to only higher b-values.

Despite being able to correct for differing session-to-session temperatures, the limited temperature resolution leaves a residual uncertainty in the ground-truth ADC measurements, increasing as ground-truth ADC increases, which drives some of the spread in ADC_bias_, repeatability and reproducibility. Conversely, the temperature accuracy which can be achieved using an ice-water phantom is fractions of a ^o^C, meaning this uncertainty is negligible [24]. This is an important consideration for future studies using the room temperature DWI phantom.

Despite this, it is possible to make assessments of ADC_bias_ and RC_short_, under the assumptions that temperature-related ADC uncertainty is uniformly distributed about the measured temperature and temperature changes within a session are likely to be small.

Long-term repeatability and reproducibility are difficult to directly compare to ice-water phantom measurements due to the temperature effects described above. As such, the 95 % limits of agreement from the ADC_bias_ calculations for each session for individual scanners and all scanners combined (Fig. 3) were used to provide a measure of long-term repeatability and reproducibility. While this measure accounts for the ADC variation due to session-to-session temperature variation, it still contains the uncertainty from the 1 °C temperature resolution (Fig. 1). With this effect included, long-term repeatability for scanners 1, 2, 3 and 5 fall within the 6.5 × 10^−5^ mm^2^ s^−1^ limit set out by the QIBA profile [7] while the scanners 4 and 6 fall slightly outside it; for reproducibility 95 % limits of agreement for the combined scanner session ADC_bias_ fall marginally outside this value. However, note that these values were calculated from all vials, not just the centre vial as specified by the QIBA profiles, and in particular reproducibility is considerably smaller than that determined by van Houdt et al. This reflects the fact that the scanners and sequence we tested are more similar than in the van Houdt et al. study which tested a range of scanner manufacturers and models, including 1.5 T and 3 T scanners [8].

To put these results in context, we must compare them to expected ADC changes throughout treatment. Radiotherapy-induced ADC changes of 0.2 to 0.3 × 10^−3^ mm^2^ s^−1^ have been measured in cervical tumours [12,25]. Such changes are greater than the repeatability coefficients estimated in our study, indicating that the sequence evaluated is a promising candidate to measure cervical ADC changes in multi-institution longitudinal trials. However, within-patient repeatability coefficients in-vivo are expected to be higher than in a phantom, so this must be assessed within any study, with e.g. pre-treatment double-baseline measurements [7], to determine if any intervention produces a real change.

Our results suggest that it is realistic to take a pre-existing clinical sequence, transfer it to a compatible scanner from the same manufacturer, and expect ADC measurements to be repeatable and reproducible. This could greatly simplify set-up and administration of multi-institution trials involving quantitative ADC measurements by reducing the need to develop bespoke sequences and perform long-term multi-institution quantitative ADC QA. While this was possible to show because we tested scanners from a single manufacturer, this was a limitation of the study, as it is not possible to extend this assessment to scanners from other manufacturers. This could be explored in future work.

In conclusion, the use of a standardised DWI sequence across multiple institutions provides ADC values which are accurate and repeatable with low measurement error, as measured in this work with a room-temperature DWI phantom. The sequence met the QIBA profile standards for accuracy (ADC bias), isocentre ADC error estimate and short-term repeatability.

While the room temperature phantom makes multi-institution quantitative ADC quality assurance logistically more practical, limited temperature resolution introduces uncertainties and makes assessment of long-term repeatability and reproducibility challenging if temperature is not precisely controlled.

## Declaration of competing interest

The authors declare that they have no known competing financial interests or personal relationships that could have appeared to influence the work reported in this paper.
