# Supplementary material for: Multi-institution longitudinal apparent diffusion coefficient measurements in a diffusion weighted imaging phantom at room temperature
Source: Phys Imaging Radiat Oncol. 2025 Jul 22;35:100814. doi: 10.1016/j.phro.2025.100814 (PMC12314170; doi:10.1016/j.phro.2025.100814)
Supplement: Supplementary Data 1 [file mmc1.docx]

# **Supplementary Material**


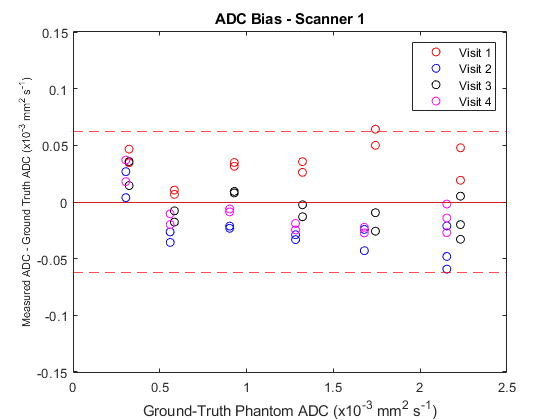


**Figure S1: ADC bias plot for scanner 1.**


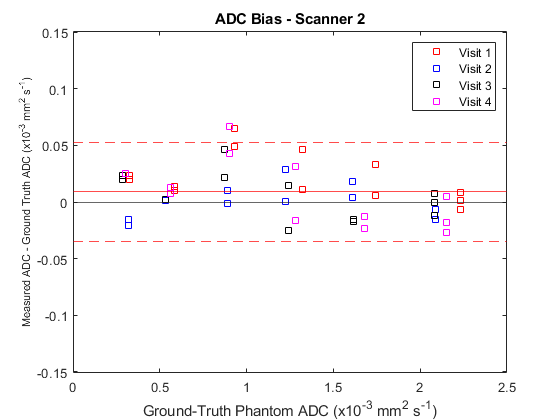


**Figure S2: ADC bias plot for scanner 2.**


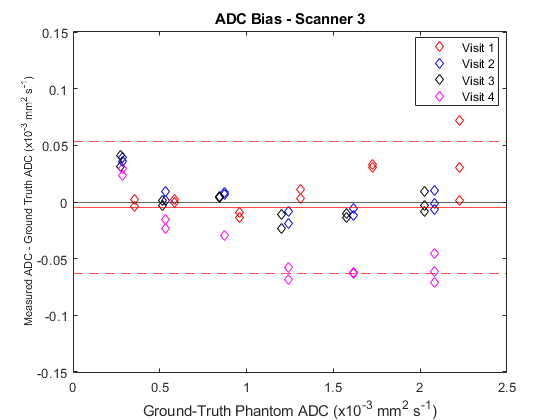


**Figure S3: ADC bias plot for scanner 3.**


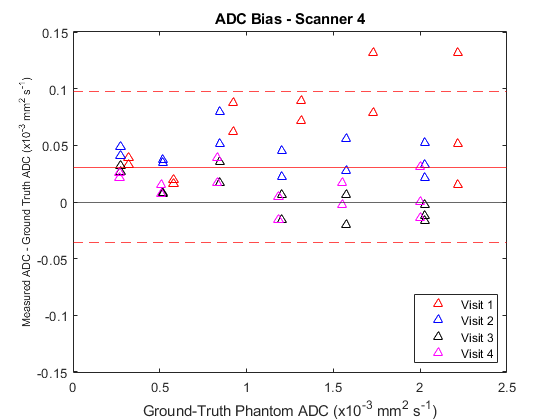


**Figure S4: ADC bias plot for scanner 4.**


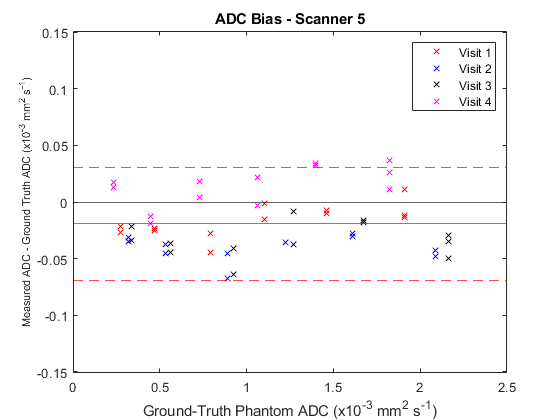


**Figure S5: ADC bias plot for scanner 5.**


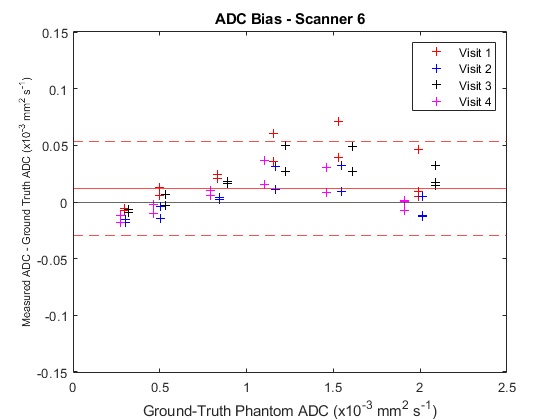


**Figure S6: ADC bias plot for scanner 6.**


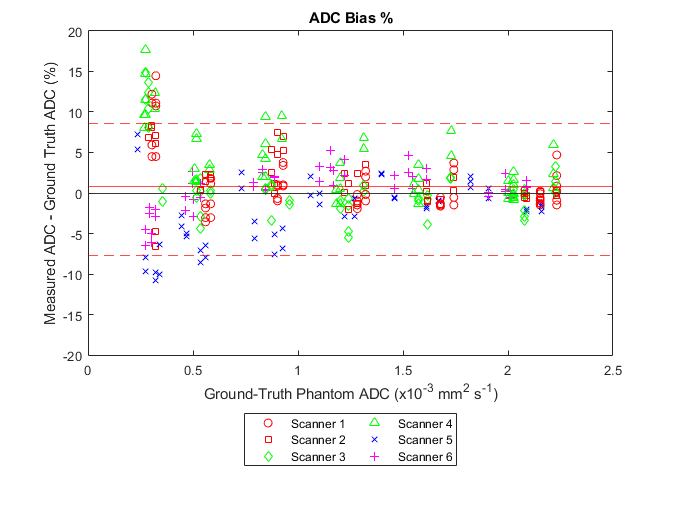


**Figure S7: Combined percentage ADC bias plot. ADC bias is plotted as a percentage of the temperature corrected ground-truth value.**


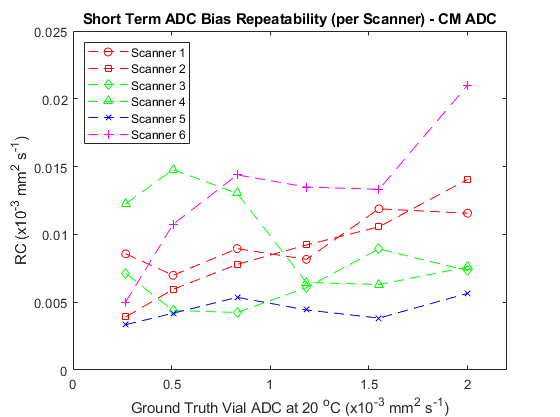


**Figure S8: Contributions of the different vials to the short-term repeatability of each scanner. For simplicity, and to allow comparison between scanners, the vial diffusion coefficients have been normalised to 20 ^o^C.**
